# Review of Recent Publications in Medical Zoology

**Published:** 1891-12

**Authors:** C. W. Stiles

**Affiliations:** Washington, D. C.


### REVIEW OF RECENT PUBLICATIONS IN MEDICAL ZOOLOGY.

By C. W. Stiles, Ph. D. Washington, D. C.

*General:*

Dr. O. Deftke. Die Entozoen des Hundes II. (Archir für wiss. u. prakt. Thierheilkûnde, xvii. 1891. p. 253-289.) In this article, published after the author's death, we find some very interesting and important statistics in regard to the internal parasites of dogs, in Berlin, Prussia. Two hundred dogs, killed at the veterinary school, were examined for parasites. The general results of the investigation are as follows:

1. The following species of internal parasites are known to exist in dogs: *Protozoa:* Cercomonas intestinalis, (Lamb); Coccidium perforans (R. Lkt); Coccidium bigeminum (Stiles); (see below). *Vermes:* Distoma campanulatum (Ercolani); D. felinum (Rivolta); D. conjunctum (Cobbold); D. echinatum (Generali); Hemistomum alatum (Diesing); Bothriocephalus latus (Bremaer); B. cordatus (Leuckart); B. serratus (Dies.); B. fuscus (Krabbe); Taenia echinococcus (v. Siebold); T. cucumerina (Bloch); T. marginata (Batsch); T. serrata (Goeze); T. coenurus (Kuchenm); T. Krabbei (Moniez); T. lineata (Goeze); T. serialis (Baillet); Echinococcus polymorphus (Hartmann); Cysticercus cellulosae (Rud); C. pisiformi (Zeder); Ascaris marginata (mystax) (Rud); Oxyuris vermicularis (Rud); Eustrongylus gigas (Dies.); Strongylus canis bronchialis (Osler); S. vasorum (Baillet); S. canium (Erc.); Dochmius duodenalis (Dubini); D. trigonocephalus (Rud.); D. stenocephalus (Rud.); Trichina spiralis (Owen); Trichocephalus depressiusculus (Rud.); Trichosoma plica (Rud.); Filaria immitis (Leidy); F. sanguinolenta (Schneider); F. medinensis (Ginelin); F. trispinulosa (Gescheidt); F. bronchialis (Blmbrg); Nemacodeum canis familiaris (Warren); Ver nématoide du rein du chien (Vûlpin); Echinorinco del cane (Perroncito); E. grassi (Grassi). *Arthropoda:* Pentastomum taenioides (Rud.); Larva of musca cadarerina; Sarcophaga çarnaria; S. hemorrhoidalis; Necrodes litoralis.

2. The dogs of Berlin are comparatively free from Entozoa.

3. Taenia marginata, T. serrata and T. coenurus were found to be the most frequent.

4. T. echinococcus and Pent. taenioides were also quite frequent.

5. The following dogs were most frequently infected: *a.* Large races, hounds (Doggen); *b.* one year old dogs and very old dogs; *c.* the males; *d.* poorly nourished dogs.

One of the parasites mentioned is coccidum perforans Lkt., frequently found in the intestines of the rabbit. I have, myself, examined a large number of dogs but have never found this species. Ralliet and I found Coc. bigeminum Stiles '91, very prevalent in Parisian dogs and it is not impossible that Deffke has mistaken this species for C., perforans. If such, however, is not the case, I can add one more parasite to Deffke's list.

The statement that poorly nourished are more frequently infected than others is possibly open to criticism. The presence of the parasite will undoubtedly explain the poor condition of many of the dogs, in other words the parasites are the direct cause of the apparently poor nourishment of the animals.

*Protozoa:*

Dr. Anton Sticker: Die gefahren der protozoen der Schlachthiere fur den Menschen (Archir f. animalische Nahrungsmittelkunde, VI., 1891, No. 2, p. 18-20; 6-7, p. 85-86; 10, 131-132). In the above named article Dr. Sticker brings forward no original results, but in a neat review of the work of other investigators, he describes the development, so far as known, of some of the microscopic parasites, in such a manner that the necessity of further original work on the subject will be apparent to the large class of people into whose hands his article must necessarily fall. He describes briefly the Coccidia, the Sporidia (Micro-Sarco, and Sarcosporidia) and Haemogregarina (falsely called by him the gregarina.)

In one place we read, "the gregarina belong to the true blood-parasites, which undergo their development inside the red blood corpuscles," etc., p. 133. This should undoubtedly read "*to* the gregarina belong, etc.," since the term gregarina, as used by the zoologists, includes a vast number of parasitic protozoa which develop in the intestines and other organs of animals, such for instance, as Clepsidrina blattarum in the intestinal canal of the roach, Monocystis agilis in the testes of the earth-worm, etc. The term Haemogregarina has been employed by many authors to designate the parasites of the red-blood corpuscles.

Dr. Sticker calls special attention to the important work done by Dr. Theobald Smith on the blood parasites in Texas fever.

Dr. Smith's discoversies in Texas fever and Prof. Laserau's discovery of the blood parasites in malaria, are described by Sticker as the most important discoveries yet made on the subject of pathogenic protozoa.

It occurs to me that Dr. Sticker lays altogether too much stress upon the dangerous character of the small cysts found in the muscular fibres of various animals and known as Sarcosporidia. So far as is yet known, these parasites are perfectly harmless to man. To prove this fact, the late Prof. Cobbold, ate a portion of meat containing thousands of these spores. Sticker dwells at some length upon the negative results obtained by Dr. L. Pfeiffer, from feeding meat infected with sarcosporidia to various animals. I have found these sarcosporidia very frequent in American and French animals, (pigs, sheep, cattle.) From April 1, to April 15, 1891, out of twenty-four sheep which I examined at the Pasteur Institute in Paris, I found twelve infected with Sarcocystis tenella and two with Gigantea. Numerous experiments followed for several months, gave only negative results, and have led me to the belief that the so-called semi-lunar disc must undergo some further change outside the body of the host, before it can infect a new animal. From hundreds of cases examined I have never found anything to support the view advanced by a certain German author, that a large number of individuals can become encysted together—in other words that some of the cysts result from a double or multiple infection. On the contrary, I believe that every cyst develops from a single spore. The authors have been somewhat divided as to whether the cyst wall is ciliated, pierced with pores or finely annulated. The cuticle of all the cysts I have examined were very finely annulated. If care is taken to isolate the sarcosporidia from the muscle fibre, the minute lines of annulation on the cyst wall are very distinctly seen. Caustic potash brings them still more clearly to view. In the middle portion of the cyst the lines run at right angles to the longitudinal axis of the parasites. At the two extremities where the cyst wall is somewhat thicker, the lines incline V-shape towards the median line.

Dr. Pfeiffer has laid great stress upon the reaction of the semilunar discs with Essin, but I have not been able to confirm his results. Further, a number of authors report that they have seen spontaneous movements of these bodies; I have studied the discs for hours at a time in bouillon, normal salt solution, water, and aqueous humor taken from sheep's eyes, both in the warmed chamber and at ordinary temperature but I have never yet been able to convince myself of any spontaneous motion of the bodies in question. I have seen movements, even such movements as L. Pfeiffer describes, but they were certainly caused by outside influences such for instance as the movement of the liquid under the cover-glass, etc. The body which is called the nucleus by many authors, is according to my observatton a vacuole. Generally only one vacuole is present, occasionally two are seen; they fail to respond to typical nuclear stains, methyl-blue, etc.

For full bibliography of the Sporozoa, to which all the animals treated in Dr. Sticker's article belong, (see Pfeiffer, Zeitschrift f. Hygiene, R. Blanchard, Bull, d. l., Soc. Zool. d. France, 1887, and Laserau, Du Paludisme et de son Hématozoaire 1891).

*Vermes :.*

Deftke, Die Entozoen des Hundes (mit 2, Tafeln), I. (Arch. f. w. u. pr. Thierheilkunde, 1891. xvii., p. 1-60.) Deffke compares minutely, both anatomically and histologically the three tape-worms, Tænia marginata, T. serrata and T. cœnurus. The work is well done, the description very clear and the figures finely executed. From his work, I will give only the following comparison of the three forms, which will enable one to easily distinguish the species from one another :

| | Taenia Marginata. | T. Serrata. | T. Coenurus. |
|---|---|---|---|
| Size | 1.5—5 m. long, the longest of the three species. | 60 cm.—2 m. | 40 60 cm., the shortest of the three species. |
| Head | Kidney form, 1 mm. in diameter. | 1.3 mm. in diameter. | Pearshape 0.8 mm. in diameter. |
| Hooks | Slender, 36-48 in number. | Largest of the three species, 38-48 in number. | short, stout, 28. |
| Neck | Not prominent. | Prominent | Not very prominent. |
| Caudal edge of segments | Extends *slightly* over the next following segment, like a cuff. | Prominent. Gives a saw-like appearance to the worm. | Not as prominent as in T. serrata. |
| Size of segments | 10-15 mm. by 4-5 mm. | 8-10 mm. by 3-4 mm. | 5-8 mm. by 3 mm. |
| Number of testes | About 600. | About 400. | About 200. |
| No. of branches to uterus | 5-6 (8) each side. | 8-10 each side. | 18-26 each side. |
| Eggs | Oval 38.5 u by 34.5 u. | 37.5 u by 32.5 u. | 35 v by 30 v. |
| Larve | Cysticercus longicollis. | Cyst. pisiformis. | Coenurus cerebralis. |
| Intermediate Host | Sheep, cattle. | Rabbit. | Lambs, calves. |

*M. G. Neumann.* Observations sûr les Ténias dû Moûton (Soc. d'Hist. nat. de Toulouse 18 Mars. 1891).

Prof. Neûmann gives the following table to aid in the determination of the tapeworms of sheep.

*Two genital pores in each segment.*

| | | | | |
|---|---|---|---|---|
| I. | *A.*— | Posterior border of segments fringed.............. | 1 | *T. fimbriata.* |
| | *B.*— | Posterior border of segments slightly undulated.. | | |
| | | *a.* Segments broad (10-25 mm.), transparent, always much wider than long......... | 2 | *T. Expansa.* |
| | | *b.* Segments thick, opaque, becoming longer than wide (10 mm. or more long)...... | 3 | *T. alba.* |
| | | *c.* Segments thick, opaque, always wider than long.............................. | 4 | *T. Benedeni* |

*One genital pore to each segment.*

| | | | | |
|---|---|---|---|---|
| II. | *A.*— | Ripe segments longer than wide.................... | 5 | *T. Vogti.* |
| | *B.*— | Segments always wider than long................... | | |
| | | *a.* Ripe segments 5-10 m.m. wide............ | 6 | *T. orilla.* (T. giardi.) (T. aculeats.) |
| | | *b.* Ripe segments 1-2 mm. wide............... | | |
| | | *b'* Opaque in the median line....... | 7 | *T. centripunctata.* |
| | | *b''* Transparent in the median line. | 8 | *T. globipunctata.* (T. oripunctata.) |

*Prof. Railliet.* Invasion du Foie et du Poumon, chez un Porcelet, par un nombre immense de Larves du *Tænia marginata* in Recueil de Médécine, Vétérinaire publié a l'ecole d' Alfort viii. No. 14, 1891. Pp. 370-373. Prof. Railliet exhibited to the Veterinary Society portions of the lungs and liver of a two-months old pig, heavily infected with cysticercus tenuicollis, (the larval form of tænia marginata, one of the dog tape-worms). The cysts measured 5-6 mm. long by 1-2 mm. wide, and were 15-20 days old. The pig had swallowed a portion of a tape-worm passed by some dog. Similar cases of infections of pigs by this cyst have been reported by R. Leuckart and Zschokke: In all cases the animals died of peritonitis and pleurisy caused by the emigration of the young cysticerci which migrated from the lungs and liver in order to complete their larval development in the serous portions of the body

The cases cited in this review add new evidence to the importance of keeping our domestic dogs free from parasites if we wish to avoid introducing certain parasitical diseases among our flocks or even into our families. We have been wont to look upon "old dog Tray" as the most faithful friend we have; yet with all his faithfulness, he is the constant source of infection of a number of parasites. A little extra care in keeping the dogs clean and free from tapeworms, etc., will not only increase his usefulness but will sometimes prevent heavy losses in our live stock. Tænia echinococcus, a small tapeworm, Tænia cœnurus—the adult form of cœnurus, and Pentastomum (Singuatula) tænioides, a worm-like arachnoid which lives in the nasal cavities of dogs—are of rare occurance in this country; probably the few cases of these parasites which have been reported in the United States were either imported or came from recently imported stock.

Persons importing dogs from Europe, especially from Russia, Germany and Scandinavia, or from Iceland, should make sure that the animals imported do not harbor these parasites; in this way, we can prevent the importation of the corresponding diseases. A simple microscopic examination of the fæces will generally determine the presence or absence of Tænia echinococcus or Tænia cœnurus, and if the slime of the nose is examined microscopically, we can generally determine whether Pentastomum tænioides is present, their eggs can be discovered in the microscopical preparation. In which case, the dogs should be subjected to a thorough medical treatment and the fæces burned or still better, the dogs should be killed and burned in toto.

