# A Correction

**Published:** 1892-02

**Authors:** 


### A CORRECTION.

Owing to a severe illness I was unable to read the proof of my last review, so take the liberty now of correcting a few typographical errors, lest some one may be bothered in looking up the references: P. 691, 19, pisiformis in place of pisiformi; 27, Nematoideum in place of Nemacodeum: 692, 28–29, Myxo in place of Sarcosporidia; 693, 1, Laveran in place of Laserau; 38, Eosin in place of Essin; p. 694, the measurements of the eggs are given in micromillimeters; p. 695, T. ovilla, T. orilla; T. aculeata, T. aculeats; T. ovipunctata, T. oripunctata; Linguatula, Singuatula.

